# Social Insurance Literacy Among the Sick-listed—A Study of Clients’ Comprehension and Self-Rated System Comprehensibility of the Sickness Insurance System

**DOI:** 10.1007/s10926-023-10166-8

**Published:** 2024-01-12

**Authors:** Elin A. Karlsson, Mattias Hellgren, Jan L. Sandqvist, Ida Seing, Christian Ståhl

**Affiliations:** 1 Department of Health, Medicine and Caring Sciences, Division of Society and Health, Unit of Public Health, Linköping University, 58183 Linköping, Sweden; 2 Department of Behavioral Sciences and Learning, Linköping University, Linköping, Sweden; 3 Department of Health, Medicine and Caring Sciences, Division of Prevention, Rehabilitation and Community Medicine, Linköping University, Norrköping, Sweden

**Keywords:** Social insurance system, Comprehensibility, Legitimacy, Sick leave, Perceived justice

## Abstract

**Introduction:**

Sickness insurance systems and their processes have been studied in terms of transparency, comprehensibility and fairness, highlighting the importance of just procedures that make sense to clients. Related research demonstrates differences between groups of clients, pointing towards a social gradient. The concept of social insurance literacy and the Social Insurance Literacy Questionnaire (SILQ) was recently developed and serves as a measure for client’s ability to obtain, understand and act on information in a sickness insurance system, relating to the comprehensibility of the information that the system provides.

**Objective:**

The purpose of this study was to investigate social insurance literacy among clients on sick leave and its associations with perceived justice, being granted sickness benefits and background factors.

**Methods:**

This was a questionnaire study with clients on sick leave in Sweden. In the selection process 3993 clients were invited, of which 1173 recently had their sickness benefits withdrawn. Those who answered the SILQ (n = 1152) also answered a perceived justice measure and accepted sharing register data from the Swedish Social Insurance Agency. Data were analyzed through regression analysis.

**Results:**

The findings demonstrate that clients’ perceptions of system comprehensibility and the status of their sick leave case was significantly associated with perceived justice, and being granted sickness benefits, while their individual abilities to obtain, understand, and act on information had lesser influence.

**Conclusions:**

The system’s ability to provide understandable information seems more important than clients’ abilities to comprehend it. From a client perspective, a just system seems to be related to their experiences of the sick leave process (i,e., whether they had an ongoing or closed case) rather than their skills to obtain the correct information.

## Introduction

In this study, we investigate the concept of social insurance literacy in relation to clients on sick leave. The importance of clients’ own understanding of how the sickness insurance system works is essential and becomes even more prominent in a restrictive system as the outcomes have major impacts upon their lives (i.e., being granted or denied sickness benefits). The Swedish sickness insurance system has undergone major changes in recent decades with the implementation of stricter activation principles. The number of people who have had their sickness benefits withdrawn has increased sharply in recent years; during the period 2015–2020, the withdrawals of sickness benefits increased dramatically [1]. The importance of clients’ understanding and acceptability of procedures they take part in is highlighted in different social insurance systems in the western countries. For instance, clients’ understanding and acceptability of procedures may influence their willingness to participate in subsequent interventions [2]. But when examining clients’ perceptions of these matters it is relevant also to adhere to the clients’ possibilities to understand the sickness insurance procedures and the comprehensibility of the information that is provided to them by the system, hence not only emphasizing individual skills but also communications skills of the system representatives. Previous research demonstrates that clients’ strategies to argue their case are more or less successful in affecting their sick leave process, depending on whether they argue matter-of-factly or emotionally [3]. Further, the fairness and acceptability of procedures within sickness insurance also seems to be dependent on the communication skills of system representatives [4]. This raises questions upon the influence of clients differing abilities on sickness insurance procedures, and legal security and fairness of the sickness insurance system.

Few clients seem to have the basic knowledge of how social insurance systems function and what rights they have, which aggravates the possibility of making informed decisions and guarding their rights [5]. Previous research also demonstrates differences between groups of clients on sick leave, for instance regarding whose eligibility for sickness benefits are questioned and who receives vocational rehabilitation interventions or not [6–11]. The research described above was the starting point for this study, in which we saw a need to investigate whether interactions in the sickness insurance system may influence clients’ possibilities to receive sickness benefits (see hypothesis 2) and their perceptions of justice (see hypothesis 3).

Literacy is a well used term that occurs in different contexts aiming to explain clients understanding of a specific context or phenomena and how this affects different outcomes and considered suitable for the phenomena that we aimed to study. Examples of such concepts are health literacy, financial literacy, health insurance literacy, legal capability and social security literacy [12]. Health literacy is a well-established concept with multiple measures developed over the past decades, commonly defined as “personal, cognitive and social skills which determine the ability of individuals to gain access to, understand, and use information to promote and maintain good health” [13, p. 264]. However, health literacy literature tends to define health literacy broader than it is being measured, thus risking emphasizing client’s individual responsibility, and overlooking structural and social issues. Research on health literacy has demonstrated associations between having low health literacy and being male, having lower education, young age, and single living with children [14]. This is similar to other health literacy research demonstrating a social gradient.

In relation to a sickness insurance context, there was a need for a concept that recognized the interactions between individuals and social insurance systems, and how this relates to the social resources of individuals and the communication strategies of systems. This gave rise to the concept of social insurance literacy. A key framework for our work with the social insurance literacy concept is the social determinants of health [12]. This framework is focused on structural differences between groups in relation to their social status, including the conditions in which people are born, grow, work, live and age, and economic policies and systems, social norms, social policies and political systems that affect these conditions [15]. The concept of the social gradient is particularly useful in this context as it highlights how health is closely linked to clients’ income and education, where differences are seen on a continuous scale and each step on the ladder provides better health [16]. Relating clients’ understanding of the sickness insurance system to how comprehensible they consider the system to be, and to perceived justice, is based on our literature search, and is new.

### Social Insurance Literacy: Previous Research

A project was initiated aiming to develop the concept of social insurance literacy (SIL), where a scoping review of definitions and operationalizations of SIL and similar concepts was performed [12]. The theory behind the concept has its roots in sociology, social medicine and public health, such as the social determinants of health that recognize structural differences between groups of people in society. Literacy in this context is not considered a skill simply possessed by the client but a dynamic relationship between the client and the system, where a social gradient likely has an impact on the client’s possibilities to guard his/her rights (see hypothesis 1). The system can, for instance, facilitate clients’ understanding of processes within the system by providing understandable and individually adapted information. SIL is defined as “*the extent to which individuals can obtain, understand and act on information in a social insurance system, related to the comprehensibility of the information provided by the system*” [12, p. 1783]. The content of the concept includes areas such as individuals’ ability to obtain, understand, and act on information regarding how to contact, navigate and deal with decisions and appeals within the sickness insurance system. It also includes the system’s comprehensibility and the system’s perceived ability to provide accessible, understandable and transparent information [12].

The focus of this study is on the importance of clients’ understanding of processes within the sickness insurance system that they are involved in, and how this may affect how just they consider the system, its processes and its outcomes. Further, this study focuses on clients’ possibilities to receive sickness benefits depending on what understanding of the system that they have. The purpose of this study was to investigate social insurance literacy among clients on sick leave and its associations with perceived justice, being granted sickness benefits and background factors.

This study had three hypotheses (H1-3), derived from research of similar literacy concepts and other studies of interactions in sickness insurance systems:

H1) The level of social insurance literacy is related to background factors, following a social gradient.

H2) A higher level of social insurance literacy is related to a higher probability to be granted sickness benefits.

H3) A higher level of social insurance literacy is related to higher perceived justice in encounters with the sickness insurance system.

## Methods

### The Swedish Sickness Insurance Context

Within the Swedish sickness insurance system, the Swedish Social Insurance Agency (SIA) has a central role of being a public authority with the primary task of assessing clients’ eligibility for sickness benefits and coordinating vocational rehabilitation. The workers at the SIA performing the formal assessments of clients’ reduced work ability and eligibility to sickness benefits will hereby be referred to as case managers. The assessment of eligibility for sickness benefits is performed using a reductionistic approach where the authority focuses on medical pre-requisites to work rather than taking contextual, individual and social factors, such as age and workplace factors, into consideration [17, 18]. Assessments within the sickness insurance system as a part of the exercise of authority, take place in different forms and have a major impact on the client’s case and their possibility to receive sickness benefits. Examples of such assessments within the Swedish system is the treating physician’s initial assessment of whether or not to write a medical certificate, the case manager’s assessment of whether or not to accept the treating physician’s medical certificate and to grant the client sickness benefits, and the documented assessment by insurance medical evaluation units in healthcare after a client’s participation in an insurance medical evaluation.

Eligibility for sickness benefits exists for up to 90 days if the client is unable to do their regular work or any other temporary work that the employer can provide. After these 90 days, and up to 180 days, eligibility exists if the client cannot do their regular work, or any other work provided by the employer. From day 181, eligibility criteria become stricter as the assessment is carried out in relation to any potential work in the regular labor market [11]. Case managers tend to foster a gatekeeper function [19] in which assessments play a central role. When the case manager considers withdrawing a client’s sickness benefits, the client first receives a letter with information from the SIA about the preliminary withdrawal and are given the opportunity to submit supplementary material within two weeks, before their case-manager makes the formal decision on whether or not to grant sickness benefits [20].

### Study Procedures

An invitation to participate in this study was sent in September 2020 by mail to 3993 clients in Sweden who were on sick leave. This distribution of invitations was managed by a printing service at Linköping University, who also were responsible for collecting the answered questionnaires and to send reminders. The invitation included information about the study, that participation was voluntary, and that the data would be treated with confidentiality. This first invitation also included a link to a web questionnaire with a personal login. Respondents had the opportunity to log in an unlimited number of times to complete the questionnaire. The first invitation was answered by 586 clients. A first reminder was sent by mail in October 2020 and included an information letter along with the SILQ-questionnaire on paper, resulting in another 482 responses. The second reminder was sent in November 2020 and resulted in another 89 responses. The web questionnaire closed at the turn of the year 2020/2021 but paper questionnaires kept coming until August 2021. All paper questionnaires were read manually by the first author (EK) to identify errors in the completion of the questionnaire that would need to be managed. This was discussed with the other authors, resulting in the exclusion of five respondents due to high item nonresponse. Further, if a respondent marked two adjacent response options or between two options in the paper questionnaire the answer was randomized into one of the answers. This was performed for a total of 15 of the respondents, of which some had several questions that needed randomization. All other types of misreporting were handled as missing values. In total, this study consisted of 1152 respondents, of which 691 answered the SILQ on the web and 461 in paper, resulting in a 28.9% response rate.

### The Selection of Respondents

In this study, we were specifically interested in recruiting those clients who were assessed towards any potential work available in the regular labor market, rather than to a specific work. This commonly occur from day 181 in the sick leave spell in the Swedish sickness insurance system, but for unemployed clients this applies from the very beginning of the spell. This represents a stricter assessment often associated with an increase of withdrawals of sickness benefits [11] that may require both skills from the client to comprehend as well as abilities of the system representatives in clarifying the steps taken. Thus, the inclusion of respondents was directed towards clients being assessed in this manner in their sick leave spell. The population of clients on sick leave are generally between 18 and 65 years old, but this was also an inclusion criterion for this study.

Regarding exclusion, managing the Swedish language enough to be able to answer the questionnaire was required since the questionnaire was only provided in Swedish.

To facilitate analyses of the group of clients who had their sickness benefits withdrawn, the sample was stratified so that 1173 of the clients had received a recent withdrawal of sickness benefits (hence had a closed sick leave case at the time of the recruitment process) and 2820 had not (hence had an ongoing sick leave case). This selection was performed by the SIA, who randomly selected clients who fulfilled the criterions above and provided the researchers with contact details.

### Non-Response Analysis

A non-response analysis was performed, in which an equal distribution regarding gender was demonstrated in both groups of respondents and non-responders. However, the respondents are to a slightly larger extent female compared to the non-responders (71% versus 63.2% with an ongoing sick leave case and 73.5% versus 61.9% with a closed case). Further, educational level and age was also somewhat higher among the respondents compared to the non-responders, and a smaller proportion of those born in other countries than Sweden responded to the questionnaire. In summary, the non-response analysis demonstrated overall expected patterns in terms of women and those with higher educational level generally having higher response rates in questionnaire studies.

### Measures

An instrument to measure SIL was previously developed, called the Social Insurance Literacy Questionnaire (SILQ). The SILQ was developed to function both on paper and online in a simultaneous process aiming at a similar appearance with the same headlines, layout and overall structure. The SILQ has recently been validated through Rasch analysis in terms of construct validity and internal consistency reliability [21] leading to the exclusion of variables that did not fit into the Rasch model or were difficult for respondents to answer. This present study is the first empirical study using the SILQ.

The SILQ-SE consists of 23 variables (see Appendix 1), relating both to the clients’ self-rated ability and how they perceived the comprehensibility of the sickness insurance system. For this study, the SILQ was divided into four subcategories, of which three represent the clients self-rated ability to take necessary contacts, to navigate through the system and to deal with decisions, and the fourth subcategory represents their perceptions of the information provided to them by the system regarding transparency and comprehensibility. Hence, the four subcategories were: (1) the individuals’ ability to obtain information, (2) their ability to understand it, (3) the ability to act on this information, and (4) the perceived system comprehensibility. For the purpose of this study, we used the four SIL subcategories as an explanatory model for the concept, as parameters relevant for SIL.

The items in the SILQ questionnaire were previously analyzed with Rasch analysis to get a subset of items with good psychometric properties [21]. Rasch analysis is a psychometric technique specifically developed for improving the precision in the construction of measures and, for instance, appropriate to use for evaluating an instrument’s ability to measure a theoretically founded variable [22]. More specifically, the Rasch analysis of the SILQ demonstrated good or acceptable psychometric properties regarding, for instance, floor- and ceiling effects, and unidimensionality. The four subcategories have been analyzed separately, hence, unidimensionality exists within each subcategory. A summative SIL score could not be estimated as the subcategories represent the individuals as well as the system’s ability and thus are too essentially different [21]. The estimated personal parameters, i.e., logit scores, from the previous Rasch study are used in this study. These are derived from both the client’s abilities and the difficulty of the questions, compared on the same scale, thus providing higher reliability than the raw scores. Since Rasch analysis provides values centered around 0, and this study uses the logit scores from Rasch, this also leads to means as presented in the results of this study being centered around 0, hence they can be either negative or positive scores. The scales in the questionnaire ranged from 1 to 4 where 1 indicated very good and 4 very poor. Correspondingly, a negative logit score indicates a higher literacy, while a positive score indicates lower literacy.

The perceived justice measure is a direct translation of another instrument, consisting of 15 variables (see Appendix 2), developed by Franche et al. [23], which was included in this study’s questionnaire and analyzed in accordance with its founders to determine a score of the client’s perceptions. The translation was made through a back translation process where the first Swedish draft was translated back to English to evaluate the accuracy of the translation. Only minor corrections in wordings were made after this process. Perceived justice is divided into four dimensions: Informational justice, Distributive justice, Procedural justice, and Interactional justice. These terms refer to the information provided (Informational justice), delivery of services or outcomes (Distributive justice), the perceptions of the processes of reaching certain outcomes (Procedural justice), and the treatment and communication from management or stakeholders (Interactional justice) [24, 25]. This scale ranged from 1 to 5 (fully agree – fully disagree), where a low score indicated higher perceived justice.

Background factors include the socio-economic characteristics educational level and self-rated financial situation, and the demographic factors age, sex, marital status, number of sick leave days, and whether the respondent was born in Sweden or not. In this study, the term “background factors” will be used to indicate both socio-economic and demographic variables. Primarily, this data was derived from the survey, except the numbers of sick leave days, which was obtained from register data and calculated in number of days on sick leave compensated by the sickness insurance system (regardless of part- or full time) for one year before and one year after the invitation to answer the survey (September 2020). Educational level was coded as either “primary/secondary school” or “college/university” in which “college/university” included a completed bachelor’s degree and above, hence no single courses. Self-ratings of financial situation ranged from “Very good” to “Very poor”. Age was used in analyses as a numerical value. Sex was coded into “female”, “male” or “other”. Marital status included whether respondents were “Living alone” or “In a relationship”, in which the latter also included those in live-apart relationships. The country of origin was coded as in “Sweden” or “Foreign”. These above-mentioned factors were related to the four SIL subcategories (i.e., Obtaining, Understanding, and Acting on information, and Perceived system comprehensibility), the four perceived justice dimensions (i.e., Informational, Distributive, Procedural, and Interactional justice), and to sickness benefit outcomes.

### Statistical Analyses

Statistical analyses were conducted by the second author (MH), using the statistical software R (ver. 4.1.1). Data were analyzed in four stages, where the initial stage was descriptive to give an overview of the material. The second stage corresponds to H1, focusing on the relationship between the SIL-measures and background factors. The SIL was analyzed through the four subcategories (Obtaining, Understanding, and Acting on information, and perceived System comprehensibility). The third stage relates to H2 and relates the SIL measures to the outcome of being granted sickness benefits or not (through the sample groups of respondents with either an ongoing case or a recently closed one). The fourth stage relates to H3, relating the SIL measures to perceived justice (Informational, Distributive, Procedural, and Interactional justice).

For each stage bivariate analyses were conducted using Welch *t*-test or Mann-Whitney U and one-way independent ANOVA for comparison, and Pearson’s *r* and Spearman’s *r* for correlation. Welch *t*-test were used as it tends to be more reliable than Students *t*-test, when the size of two sample groups is unequal [26]. Mann-Whitney U was used when normality assumptions were violated as was Spearman’s *r*. For Welch t-test and Mann-Whitney U effect size was computed, Cohens *d* and *r* respectively whereas for ANOVA omega-squared (*ω*^*2*^) was computed.

The second stage and onwards were further analyzed using regression models, OLS for the numeric SIL and justice measures (2nd and 4th stages) and logistic regression for the binary measure of whenever the respondent were granted sick leave or not (3rd stage). In the regression models all 1152 respondents were included. For the third and fourth stage of the analysis, three models were produced, one containing the individual abilities (A), one containing the system’s ability (B) and a third (C) containing all four SIL subcategories. This modeling was considered relevant as clients’ individual abilities and their perceptions of the sickness insurance system represent abilities at two different levels (i.e., individual versus system), and thus needed to be separated in two different models. For each model Aikane Information Criterium (AIC) has been computed for model comparison.

### Ethics

Study procedures and data collection has been performed in accordance with the Helsinki Declaration [27]. Respondents were informed about participation being voluntary and confidential, that they have the right to withdraw their participation at any time, and provided with contact information to the researchers (CS and EK) in case of any questions. This study was approved by the Swedish Ethical Review Authority (Dnr 2019 − 01671).

## Results

The study consisted of 1152 respondents with two sample groups, one consisting of those that had an ongoing sick leave case (*N* = 754, 65.5%) and one that had a recently closed case (*N* = 398, 34.5%). Characteristics of the respondents can be found in Table 1. While age has been reported in categories, for an overview of the characteristics of the respondents in Table 1, the numeric values have been used in analysis.

**Table 1:** Characteristics of the respondents

	Respondents N(%)
Total	1152
*Sex*	
Male	322 (28.0%)
Female	828 (71.9%)
Other	1 (0.1%)
*Age*	
20–34	164 (14.3%)
35–49	395 (34.3%)
50–59	385 (33.4%)
> 60	206 (17.9%)
*Education*	
Primary	86 (7.5%)
High school	421 (36.7%)
University	547 (47.7%)
Other	9 (0.8%)
*Marital status*	
Living alone	315 (27.4%)
Married/co-living	766 (66.6%)
Partnered, separate living	32 (2.8%)
Other	37 (3.2%)
*Self-rated financial situation*	
Very good	73 (6.3%)
Good	275 (23.9%)
Ok	355 (30.8%)
Poor	261 (22.7%)
Very Poor	185 (16.1%)
*Duration of current sick leave spell at the time of recruitment*	
No current sick leave case	215 (18.7%)
1–90 days	8 (0.7%)
91–180 days	179 (15.5%)
181–365 days	157 (13.6%)
1–2 years	359 (31.2%)
2–5 years	232 (20.1%)
5 < years	2 (0.2%)

When data are missing the numbers do not sum up to 1152. This data is mainly collected from the SILQ although sick leave spells were extracted from the register data

In comparing the sample groups with regard to background factors, except for marital status, no significant differences were found. For marital status, there is a significant difference (*Χ*^2^(1, *N* = 1152) = 7.48, *p* = 0.006, *w* = 0.08) with closed having a higher proportion (76.9%) of being in a relationship compared to those with an ongoing sick leave case (68.9%).

Means for the SIL subcategories ranged from 0.20 to -0.82, with − 0.48 for Obtain (SD = 1.76), -0.82 for Understand (SD = 1.70), -0.40 for Act (SD = 1.52), and 0.20 for System comprehensibility (SD = 1.88), indicating an overall lower comprehension of the system (which had the highest score) compared to the individual abilities Obtaining, Understanding, and Acting upon information.

Among the respondents, the median sick leave in days during the two-year period of measurement, one year prior and one year after, were 420.50. Due to the sampling groups being from those with ongoing cases (Mdn = 562.5) and those without an ongoing case (Mdn = 176.5) there is as expected a distinct difference between the groups, *U* = 18248.5, *p* < 0.001, *r* = 0.72.

### Social Insurance Literacy Related to Background Factors

The SIL was initially tested against the background factors, thus testing H1, whether there is a potential social gradient in SIL. The dichotomous variables were tested using Welch *t*-test (see Table 2). The sole numerical variable age were tested using Spearman’s’ *r* due to age showed a significant departure from normality, *W*(0.950), *p* < 0.001. No significant correlations were found between age and Understand (*r*_*s*_(1147) = -0.03, *p* = 0.265) while significant correlations were found between Act (*r*_*s*_(1142) = − 0.06, *p* = 0.037), Obtain (*r*_*s*_(1143) = -0.07, *p* = 0.014), and System (*r*_*s*_(1142) = -0.08 *p* = 0.010), though these are fairly negligible.

**Table 2:** Comparison between SIL and background factors (Welch *t*-test)

SIL	Variable	Factor	N	M	SD	*t*	*p*	*d*
Obtain	Gender	Female	828	− 0.57	1.78	− 2.60	0.009	− 0.17
		Male	322	− 0.27	1.70			
	Marital status	In relationship	798	− 0.57	1.78	− 2.72	0.007	− 0.18
		Living alone	315	− 0.25	1.73			
	Born in	Sweden	990	− 0.45	1.76	− 0.98	0.330	− 0.09
		Foreign	152	− 0.61	1.79			
	Education	Primary/secondary	561	− 0.29	1.80	− 4.23	< 0.001	− 0.26
		College/University	547	− 0.73	1.69			
Understand	Gender	Female	828	− 0.91	1.71	− 2.88	0.004	− 0.18
		Male	322	− 0.59	1.68			
	Marital status	In relationship	798	− 0.87	1.70	− 1.64	0.102	− 0.11
		Living alone	315	− 0.69	1.72			
	Born in	Sweden	990	− 0.80	1.69	− 0.37	0.715	− 0.03
		Foreign	152	− 0.86	1.77			
	Education	Primary/secondary	561	− 0.58	1.61	− 5.38	< 0.001	− 0.35
		College/University	547	− 1.12	1.75			
Act	Gender	Female	828	− 0.45	1.55	− 1.93	0.054	− 0.12
		Male	322	− 0.26	1.43			
	Marital status	In relationship	798	− 0.45	1.53	− 1.80	0.070	− 0.12
		Living alone	315	− 0.27	1.54			
	Born in	Sweden	990	− 0.37	1.53	− 0.95	0.343	− 0.08
		Foreign	152	− 0.49	1.45			
	Education	Primary/secondary	561	− 0.23	1.42	− 3.83	< 0.001	− 0.23
		College/University	547	− 0.58	1.59			
System	Gender	Female	828	0.18	1.83	− 0.58	0.562	− 0.04
		Male	322	0.26	2.01			
	Marital status	In relationship	798	0.15	1.89	− 1.27	0.206	− 0.08
		Living alone	315	0.31	1.89			
	Born in	Sweden	990	0.26	1.84	− 1.75	0.082	0.08
		Foreign	152	− 0.05	2.04			
	Education	Primary/secondary	561	0.08	1.90	− 1.91	0.056	− 0.11
		College/University	547	0.30	1.86			

*d* = Cohens *d*

Overall relating the SIL measures to background variables show negligible to medium effects (see Table 2). For the individual measures, education stands out, overall having a higher level of education yields a lower score, thus indicating a higher literacy. For instance, illustrated by a negative mean of -1.12 for Understanding information compared to a higher mean of -0.58 for respondents with lower educational level. In addition to education, gender and marital status for Obtain, while gender for Understand, showed significant differences, though with small effects. For Act, education is the sole significant variables and for System none of the background variables are significant. Notably is that country of origin showed no significant differences over all measures.

Regression models of the SIL measures related to background variables yield similar results as of which variables are influential as the bivariate tests (see Table 3). Overall, the models indicate poor prediction of SIL with *R*^2^ values ranging from 0.019 to 0.036, although the results are in the expected direction, i.e., that higher educational level is related to a higher individual literacy. Evaluation of assumptions show no violation of multicollinearity, residual normality, homoscedasticity, or linearity. Further, as a final step in testing H1 and the potential occurrence of a social gradient, analyses of SIL and the clients’ self-rated financial situation were performed, using one-way independent samples ANOVA (see Table 4). For this item, a scale of 1–5 was used, in which 1 represents a perceived very good financial situation and 5 a very poor. In general, there is a trend where SIL increases when the perceived financial situation is rated better, hence, the better financial situation the higher SIL. The differences are significant, although the effects (*ω*^*2*^ is between ,05 and ,11) are somewhat low. Thus, the hypothesis of SIL having a social gradient have support, although the influence is marginal.

**Table 3:** OLS regression models predicting variation in SIL related to background factors

	Obtain	Understand	Act	System
Constant	− 0.140	− 0.671^**^	− 0.243	0.549^*^
	(0.292)	(0.283)	(0.254)	(0.317)
Gender	0.296^**^	0.272^**^	0.164	0.157
(Male)	(0.123)	(0.119)	(0.107)	(0.133)
Born Sweden	0.167	0.087	0.134	0.322^*^
(Sweden)	(0.159)	(0.154)	(0.138)	(0.174)
Relationship	0.257^**^	0.134	0.130	0.139
(Living alone)	(0.119)	(0.115)	(0.104)	(0.129)
Educational level	0.393^***^	0.503^***^	0.318^***^	0.237^**^
(Primary/Secondary)	(0.110)	(0.106)	(0.096)	(0.119)
Age	− 0.017^***^	− 0.012^**^	− 0.010^**^	− 0.017^***^
	(0.005)	(0.005)	(0.004)	(0.005)
*R* ^2^	0.036	0.036	0.022	0.019
*F* Statistic	7.771^**^ (5; 1047)	7.943^**^ (5; 1051)	4.636^**^ (5; 1046)	3.967^**^ (5; 1046)

*p < 0.05, **p < 0.01, ***p < 0.001. Reference category for dichotomous variables given in parenthesis, for values *B* and standard errors (SE), *B* are given, with SE *B* in parenthesis

**Table 4:** Comparison between self-rated financial situation and SIL measures (ANOVA)

Financial situation		Obtain information		Understand information		Act upon information		System comprehensibility	
	*N*	*M*	*SD*	*M*	*SD*	*M*	*SD*	*M*	*SD*
1	73	− 1.85	1.70	− 2.27	1.66	− 1.47	1.59	− 1.44	1.99
2	275	− 0.88	1.72	− 1.19	1.62	− 0.65	1.48	− 0.13	1.77
3	355	− 0.54	1.63	− 0.82	1.61	− 0.38	1.40	0.09	1.83
4	261	− 0.10	1.64	− 0.52	1.57	− 0.12	1.42	0.45	1.72
5	185	0.24	1.79	− 0.08	1.72	− 0.01	1.66	1.24	1.64
ANOVA		*F*(4, 1138) = 27,54, *p* < ,001, *ω*^*2*^ = ,08		*F*(4, 1142) = 29,69, *p* < ,001, *ω*^*2*^ = ,09		*F*(4, 1137) = 17,02, *p* < ,001, *ω*^*2*^ = ,05		*F*(4, 1137) = 35,05, *p* < ,001, *ω*^*2*^ = ,11	

*p < 0,05, **p < 0,01, ***p < 0,001

## Social Insurance Literacy Related to the Probability of Being Granted Sickness Benefits

The second hypothesis was that higher SIL increases the probability of being granted sickness benefits, assuming that these clients know how to argue their case. As noted earlier, the sample consists of two groups, one granted sickness benefits and one recently experiencing a withdrawal. The differences between these groups were tested using Welch *t*-test (see Table 5).

**Table 5:** Social insurance literacy related to sickness benefit outcomes (Welch *t*-test)

Measure	Sickness benefits	N	M	SD	*t*	*p*	*d*
Obtain	Ongoing	754	-0.63	1.75	3.89	< 0.001	0.24
	Closed	398	-0.21	1.75			
Understand	Ongoing	754	-0.99	1.69	4.75	< 0.001	0.29
	Closed	398	-0.49	1.68			
Act	Ongoing	754	-0.51	1.50	3.31	< 0.001	0.21
	Closed	398	-0.19	1.54			
System	Ongoing	754	-0.26	1.90	13.05	< 0.001	0.75
	Closed	398	1.07	1.48			

*d* = Cohens *d*

The results showed consistent significant differences between the groups, with effects ranging from medium to strong. Throughout, the ongoing group scored lower compared to the closed group, indicating a higher literacy. The results point towards that being granted sickness benefits is associated with a higher individual ability to Obtain, Understand and Act on information, as well as perceiving System comprehensibility as better.

To test the influence of SIL on being granted sickness benefits, logistic regression was used (see Table 6). Three models were produced, one containing the individual abilities (A), one containing the system’s ability (B) and a third (C) containing all four SIL subcategories. Evaluation of the models showed no violations of assumptions. To evaluate the models AUC and McFadden *R*^2^ were computed. AUC assesses the discrimination whereas McFadden *R*^2^ yield a value that is more comparable to the *R*^2^ value of OLS.

**Table 6:** Three logistic regression models for being granted sickness benefits

	Sickness benefits (1 = Ongoing)		
	Model A	Model B	Model C
Constant	0.483^***^	2.557^***^	0.920^***^
	(0.070)	(0.032)	(0.088)
Obtain	0.005		0,069
	(0.047)		(0.086)
Act	0.047		0.118 ^**^
	(0.078)		(0.083)
Understand	− 0.218^**^		0.100
	(0.076)		(0.084)
System		− 0.454^***^	− 0.511^***^
		(0.042)	(0.049)
*N*	1137	1144	1137
AIC	1454	1337	1318
R^2^_McF_	0.016	0.097	0.103
AUC	0.587	0.707	0.715
*χ* ^2^ Statistic	23.7^***^ (df = 3)	144^***^ (df = 1)	151.8^***^ (df = 4)

*R*^2^_McF_ = McFadden pseudo *R*^2^ *B* and SE *B* are given, with SE *B* in parenthesis

*p < 0.05, **p < 0.01, *** p < 0.001

While there are medium to strong effects in the difference of the measurements between those granted and those not granted sickness benefits (see Table 6), the models indicate that the SIL measures are fairly poor in predicting the variability of being granted sickness benefits, with *R*^2^ scores ranging from 0.02 to 0.10. The discrimination of model B and C is within acceptable range (Hosmer & Lemeshow, 2013) with AUC scores of 0.71 and 0.72, respectively. However, model A shows poor discrimination. These results indicate that the clients’ individual abilities are of less importance for receiving sickness benefits as opposed to System comprehensibility. Perceiving the system as comprehensible was significantly associated with having received sickness benefits. Taken together, system comprehensibility seems to be the strongest factor for predicting whenever sickness benefits are granted or not while also showing the strongest difference between the groups and influence on variability. H2 is hereby partly supported.

## Social Insurance Literacy Related to Perceived Justice in Encounters with the Sickness Insurance System

In H3, a higher level of SIL was expected to be associated with a higher degree of perceived justice. The relationship between SIL and the justice measure was initially explored using correlation analysis (see Table 7) and then using SIL measures in OLS regression (see Table 8). For the justice measures, means ranged from 2.31 to 2.70, with 2.64 for Distributive justice (SD = 1.31), 2.67 for Procedural justice (SD = 1.35), 2.70 for Informational justice (SD = 1.45), and 2.31 for Interpersonal justice (SD = 1.52), indicating that Informational justice, with the highest score, was perceived least fair, while Interpersonal justice was perceived as most fair of the justice dimensions.

**Table 7:** Correlation matrix of SIL and justice measure (Pearsons *r*)

Measure	1.	2.	3.	4.	5.	6.	7.	8.
1 Obtain	1.00	0.84	0.81	0.42	0.31	0.33	0.37	0.28
2 Understand	0.84	1.00	0.76	0.49	0.34	0.37	0.40	0.34
3 Act	0.81	0.76	1.00	0.32	0.21	0.24	0.29	0.20
4 System	0.42	0.49	0.32	1.00	0.62	0.70	0.73	0.69
5 Distributive Justice	0.31	0.34	0.21	0.62	1.00	0.74	0.55	0.55
6 Procedural Justice	0.33	0.37	0.24	0.70	0.74	1.00	0.65	0.65
7 Informational Justice	0.37	0.40	0.29	0.73	0.55	0.65	1.00	0.71
8 Interpersonal Justice	0.28	0.34	0.20	0.69	0.55	0.65	0.71	1.00

All correlations are significant at 0.001 level

**Table 8:** OLS regression for Justice measures

	Distributive justice			Procedural justice			Informational justice			Interpersonal justice		
	Model A	Model B	Model C	Model A	Model B	Model C	Model A	Model B	Model C	Model A	Model B	Model C
Constant	2.853^***^	2.557^***^	2.560^***^	2.853^***^	2.557^***^	2.560^***^	2.990^***^	2.596^***^	2.612^***^	2.566^***^	2.198^***^	2.181^***^
	(0.042)	(0.032)	(0.038)	(0.042)	(0.032)	(0.038)	(0.045)	(0.031)	(0.038)	(0.048)	(0.035)	(0.042)
Obtain	0.175^***^		0.111^***^	0.115^**^		0.047	0.142^***^		0.066^*^	0.086		0.004
	(0.047)		(0.040)	(0.048)		(0.038)	(0.050)		(0.039)	(0.054)		(0.044)
Understand	0.253^***^		-0.027	0.332^***^		0.008	0.349^***^		-0.019	0.386^***^		0.018
	(0.043)		(0.039)	(0.044)		(0.038)	(0.046)		(0.039)	(0.050)		(0.043)
Act	-0.228^***^		-0.086^**^	-0.207^***^		-0.054	-0.196^***^		-0.020	-0.265^***^		-0.084^**^
	(0.045)		(0.039)	(0.045)		(0.037)	(0.048)		(0.038)	(0.052)		(0.043)
System		0.408^***^	0.406^***^		0.472^***^	0.470^***^		0.537^***^	0.529^***^		0.520^***^	0.537^***^
		(0.017)	(0.020)		(0.016)	(0.019)		(0.017)	(0.020)		(0.018)	(0.022)
AIC	3663.1	3342.8	3294.3	3643.8	3198	3149	3842.8	3310.7	3270.6	4036.5	3579.9	3541.1
R^2^	0.127	0.345	0.361	0.150	0.432	0.447	0.169	0.484	0.493	0.117	0.413	0.422
F Statistic	54.446^***^	594.504^***^	158.508^***^	65.430^***^	850.015^***^	223.128^***^	76.162^***^	1,058.988^***^	272.623^***^	50.180^***^	799.552^***^	205.855^***^
	(3; 1126)	(1; 1131)	(4; 1121)	(3; 1111)	(1; 1116)	(4; 1106)	(3; 1125)	(1; 1130)	(4; 1120)	(3; 1132)	(1; 1137)	(4; 1127)

R^2^McF = McFadden pseudo R^2^ B and SE B are given, with SE B in parenthesis

*p < 0.05, **p < 0.01, *** p < 0.001

Overall, the relation between the individual SIL measures and the justice measures are weak to moderate with positive correlations between 0.20 and 0.40 (see Table 7). System comprehensibility on the other hand show moderate to high correlation with coefficients ranging from 0.55 to 0.71, indicating that when System comprehensibility is considered better, so is the degree of perceived justice. To test the SIL measures, OLS regression was used with three models for each justice measure, including all 1152 respondents. Model A containing the individual measures of SIL: Obtaining, Understanding, and Acting upon information. Model B containing System and the third, model C, all four measurements. Inspection of plots show no concern for violations of residual normality, homoscedasticity, and linearity. However, while all models have variance inflation factor (VIF) scores that mostly are within acceptable ranges, Obtain is consistently scoring slightly above 5 in all models, with a max of 5.12, indicating potential problems with multicollinearity. The general takeaway from the regression models is that system is the strongest predictor, with the individual being weaker. These results imply that H3 is partly supported. A sickness insurance system that is perceived as comprehensible is thus also a system that is perceived as fair.

## Discussion

This study investigated social insurance literacy among clients on sick leave, and how this was associated with perceived justice of processes within the system and being granted sickness benefits. Overall, this study indicates that both clients’ individual abilities to obtain, understand, and act on information in the system, and how they perceived system comprehensibility, were associated with sickness benefit outcomes. However, the perceived system comprehensibility was the strongest predictor. Further, a higher system comprehensibility was associated with a higher perceived justice.

### A Social Gradient in Social Insurance Systems

Navigating through a complex system is difficult for many, perhaps in particular for clients on sick leave. Another study demonstrated that clients who were on some kind of public support had lower health literacy compared to the working population, and that the clients who were unemployed had even lower health literacy than those on sick leave [14]. It would be reasonable to assume that clients with low health literacy also may have limited SIL. This study’s findings indicate a social gradient also in SIL, supporting the first hypothesis, although the predictability was relatively weak. Background factors had negligible to medium effects on the SIL subcategories (i.e., obtaining, understanding, and acting on information, and the perceived system comprehensibility), but socio-economic factors (educational level and perceived financial situation) stood out. In a study using a slightly different Dutch version of SILQ, the SILQ-NL, findings demonstrate that limited SIL is associated with living without a partner and/or being male [28]. Further, clients who lived in a more socially exposed area with, for instance, higher unemployment rates in the Netherlands rated their ability to navigate in the system higher, which may be explained with a potential learning effect from previous interactions with the sickness insurance system. Findings also highlighted that those clients who perceived the system as most comprehensible were (surprisingly) those with limited language skills, and (not as surprising) those who lived together with a partner, indicating that social support matters [28]. The differences in results may partly be explained by the Swedish and the Dutch sickness insurance system being quite different, hence contextual factors not being the same.

There may also be other aspects influencing the clients’ sick leave case, and their SIL, such as the type of diagnosis and severity or complexity of their condition, and whether their work disability is considered visible or not by the SIA, hence not being questioned. This study’s findings demonstrate that background factors have little influence on whether the clients received sickness benefits or not, but future studies could also investigate whether diagnosis may be associated with SIL, perceived justice, and sickness benefit outcomes.

### Fairness and Comprehensibility Intertwined

The results indicate that those with granted sickness benefits have a higher SIL compared to those not being granted. However, taken together, System comprehensibility demonstrated the strongest impact on sickness benefit outcomes. The second hypothesis is therefore partly supported, regarding the system dimension of SIL. Orchard et al. [29] state that a just, open and respectful communication between a sickness insurance representative and the client, as well as sufficient and clear information, is essential in order to reduce the risk of the sick leave process leading to mental illness of the client. Other studies have demonstrated that respectful encounters from case managers can facilitate the return-to-work process and enhance the clients’ self-estimated ability [30, 31]. System comprehensibility is likely depending also on other factors, such as the communication skills of system representatives, and the characteristics of the interactions between clients and their case manager. As previously mentioned, the individual abilities of SIL had weak associations to perceived justice. The system comprehensibility dimension of SIL did however have an impact, as clients who found the system understandable and logical, also to a larger extent rated it as fair. The findings indicate that system comprehensibility is influential for perceived justice, but that the client’s individual abilities are not; hence, hypothesis three is partly supported, regarding the system dimension of SIL. Thus, it is not the varying individual abilities that impact clients’ perceptions of justice, but rather the system’s ability to provide transparent and understandable information for a diverse range of clients and performing just procedures. An important implication of these findings is the matter of providing individually adapted and accessible information, as well as interactions and processes that make sense and seem just to the clients, in order for clients to perceive the system as comprehensible and just.

### Social Insurance Literacy and the Impact of Interactions in the System

SIL is a complex and elusive concept that likely is influenced by many factors which may interact in various ways. For instance, the perceived system comprehensibility is rated by clients on sick leave and reflects their experiences and understanding of the system which ought to differ substantially depending on their encounters and the information provided to them by their case manager. The precision of these kind of self-ratings can be discussed, as well as whether SIL is related also with other factors that this project has not covered, that may differ from other literacy concepts and contexts such as health literacy. System comprehensibility aspects may be more crucial in a sickness insurance context compared with health literacy, due to the structure and impact that the sickness insurance system has. For instance, a sickness insurance system is obligated to assess clients’ work ability and their right to sickness benefits, which is influenced by political demands and internal processes within the organization in order to reach certain goals and outcomes, and whether a restrictive or more generous path is currently being adopted by the system.

Further, case managers often act as sickness insurance guardians [19], as opposed to professionals in the health care system who have another role. If the clients’ abilities to obtain, understand and act on information only have a weak influence over their possibilities to receive sickness benefits, then a logical assumption would be that the processes within the system have a larger influence over the outcome. This may be interpreted positively if this means that clients have the same possibilities and are treated equitably by the system. However, it may equally be interpreted negatively if this represents the authority’s internal steering and political goals being superior to clients’ explanations and arguments in their case, implying that no matter how they act, clients are at risk of having their benefits withdrawn due to policy pressure.

In another, qualitative study [3], the characteristics of how information was communicated affected clients’ possibilities to receive sickness benefits and their perceived justice. This was demonstrated through clients arguing their right against the SIA in a formal, “fact-driven” way, being more successful in affecting their sick leave process compared to the clients who protested in a way characterized by desperation and frustration [3].

The present study did not focus on the unique characteristics of interactions between client and system, such as the relation between clients and their case manager and how thoroughly the case manager has explained procedures in the client’s case. The data collection was however carried out during a period where the system has had a restrictive approach to granting benefits, thus the client’s individual skills might not have had the same influence as they potentially could have during a more generous administration. These aspects and their influence on SIL would benefit from further studies using a qualitative design in order to gain a more nuanced description, as the concept seems difficult to fully grasp at this stage.

#### Methodological Considerations

The methodological choices made in this study will be discussed in terms of strengths and limitations. First, through the SILQ-project, several steps were taken in developing the concept and measure of SIL, including a scoping review of related concepts, a Delphi study with an international expert panel, and a Rasch study to evaluate the measure [12, 21]. Thus, this is a validated measure based on a theoretical framework, which is a strength. Further, the possibility to answer either on paper or online, was a strategy aiming at facilitating participation for a diverse range of clients and thus to increase the response rate. The SILQ was developed to function both on paper and web and these two forms were simultaneously developed aiming at a similar appearance with the same headlines, layout and overall structure, which could be considered a strength. It was considered important to be able to also include clients without access to a computer, a group who might have lower SIL. Thus, the SIL among clients who answered on paper versus on web might differ, although no such indications of the client’s comprehension of items in the paper and web questionnaire were obvious.

The response rate was somewhat low with 28.9%, which could be considered a limitation. This was to some extent expected due to the study population being clients on sick leave, hence, currently work disabled which might have caused them difficulties in filling out this questionnaire. As in most research, there is a risk that those who answer a questionnaire are the ones most interested in the topic or most capable to respond. This might have impacted this study’s results in terms of not being able to include clients who have severe difficulties or difficulties with certain areas, for instance cognitive difficulties. Thus, those clients with a lower SIL may not have been able to participate in this study and there may be an underrepresentation of clients with conditions that could have hampered their ability to respond to this comprehensive questionnaire.

The associations between the four subcategories and SIL need further investigation and the SILQ needs further evaluation in different empirical contexts. In this study, the four SIL subcategories were used as a model to explain the concept and can be considered a rough estimation of parameters relevant for SIL. These subcategories are considered prerequisites for SIL and distinguish the concept’s theoretical content. Future studies with qualitative methods could deepen and nuance the knowledge of SIL and potentially show how to fully capture it in a measure. However, with this study an important area to further investigate has emerged, i.e., the importance of system comprehensibility for clients’ perceived justice.

## Conclusions

A general conclusion from the study is that how respondents perceived the sickness insurance system’s comprehensibility had stronger associations with sick leave and perceived justice compared to individual abilities. The comprehensibility of the information provided to clients by the sickness insurance system was clearly associated with perceived justice and sickness benefit outcomes. Specifically, we found that higher ratings of system comprehensibility were related to perceiving the system as more just and a higher probability of having an ongoing sick leave case. An important task for sickness insurance institutions is to give clients with diverse prerequisites individually adapted information and offer suitable procedures, in order for people to be able to participate in their sick leave process and to perceive the system as understandable and just.

## Appendix

See Table 9 and 10.

**Table 9:** Items of the Social Insurance Literacy Questionnaire, Swedish version (SILQ)

Domain	Items (N = 23)
*System comprehensibility*	
	1. As a whole, how do you rate the Social Insurance Agency’s’ ability to offer information that you can understand?
	2. As a whole, how do you rate the Social Insurance Agency’s’ ability to make decisions within a reasonable time?
	3. As a whole, how do you rate the Social Insurance Agency’s’ ability to clearly explain the reasons for decisions?
	4. How do you think that the staff at the Social Insurance Agency succeeds in being available when you need to get in touch with them?
	5. How do you think that the staff at the Social Insurance Agency succeeds in showing that they trust you and what you tell them?
*The ability to obtain information*	
	6. How do you rate your ability to get the information you need from the Social Insurance Agency?
	7. How do you rate your ability to with help from others get the information you need (e.g., relatives, others on sick leave, health care professionals, employers, union representatives)?
	8. How do you rate your ability to get information about your possibilities to influence your sick leave case?
	9. How do you rate your ability to get information about other actors’ roles in your sick leave case (e.g., health care, employers, union representatives)?
	10. How do you rate your ability to get information about laws and regulations?
	11. How do you rate your ability to get clarifications about decisions in your sick leave case if necessary?
*The ability to understand information*	
	12. How do you rate your ability to understand how to fill in forms?
	13. How do you rate your ability to understand spoken information from staff at the Social Insurance Agency?
	14. How do you rate your ability to with help from others understand information from the Social Insurance Agency (e.g., relatives, others on sick leave, health care professionals, employers, union representatives)?
	15. How do you rate your ability to understand what information you are expected to supply to the Social Insurance Agency?
	16. How do you rate your ability to understand at what times you need to supply information to the Social Insurance Agency?
	17. How do you rate your ability to understand the laws and regulations related to your sick leave case?
	18. How do you rate your ability to understand decisions from the Social Insurance Agency?
*The ability to act upon information*	
	19. How do you rate your ability to ask questions if you need more information?
	20. How do you rate your ability to deliver information (e.g., medical certificates) to the Social Insurance Agency on time?
	21. How do you rate your ability to argue for your case by referring to laws, regulations, or certificates?
	22. How do you rate your ability to get help from others to argue for your case (e.g., relatives, others on sick leave, health care professionals, employers, union representatives)?
	23. How do you rate your ability to formally appeal decisions if you think it is wrong (e.g., to an independent tribunal or a court)?

**Table 10:** Items of Perceived Justice in accordance with the measure by Franche et al. (2009)

Dimensions	Items (N = 15)
*Distributive justice*	
	1. Overall, your compensation benefits have been fair and acceptable
	2. Considering the nature of your injury, the amount of compensation you have been receiving has been fair and acceptable
	3. Considering the nature of your injury, the length of time that you have been receiving compensation benefits has been fair and acceptable
	4. Considering your previous level of pay, the amount of compensation has been fair and acceptable
*Procedural justice*	
	5. You have been able to express your views and feelings when the SIA has made decisions about your compensation benefits.
	6. You have had influence over your compensation benefits
	7. The way that the SIA has been making decisions has not been prejudiced or biased against you 8. The SIA has been collecting correct information to make decisions
	9. The way that the SIA has been making decisions has been ethical
	10. The way that the SIA has been making decisions has been fair to you
*Informational justice*	
	11. The person from the SIA has provided you with the information you needed
	12. The person from the SIA has carefully and completely explained the way decisions are made
	13. The person from the SIA has communicated details at the appropriate times
*Interpersonal justice*	
	14. The person from the SIA has treated you in a polite manner 15. The person from the SIA has treated you with dignity and respect
